# Circular RNA circFADS2 is overexpressed in sepsis and suppresses LPS-induced lung cell apoptosis by inhibiting the maturation of miR-15a-5p

**DOI:** 10.1186/s12865-021-00419-7

**Published:** 2021-05-12

**Authors:** Xiaoyang Hong, Shuanglei Li, Jie Wang, Zhe Zhao, Zhichun Feng

**Affiliations:** 1 Pediatric Intensive Care Unit, The Seventh Medical Center, PLA General Hospital, No. 5 Nanmencang, Dongshitiao, Dongcheng District, Beijing, 100700 P. R. China; 2 Department of Cardiovascular Surgery, PLA General Hospital, Beijing, 100853 P. R. China; 3 Surgical Pediatric Intensive Care Unit, Children’s Hospital Affiliated of Zhengzhou University, Zhengzhou City, Henan Province 450018 P. R. China

**Keywords:** Sepsis, circFADS2, miR-15a-5p, Precursor, Apoptosis

## Abstract

**Background:**

Circular RNA circFADS2 plays protective roles in LPS-induced inflammation, which promotes sepsis, suggesting its involvement in sepsis.

**Methods:**

Expression of circFADS2, mature miR-15a-5p, and miR-15a-5p precursor in plasma samples from sepsis patients and healthy controls was determined by RT-qPCR. The circFADS2 expression vector was transfected in lung cells, followed by the measurement of the expression levels of mature miR-15a-5p and miR-15a-5p precursor to study the role of circFADS2 in miR-15a-5p maturation. Cell apoptosis was analyzed by cell apoptosis assay.

**Results:**

CircFADS2 was upregulated in sepsis and inversely correlated with mature miR-15a-5p, but not miR-15a-5p precursor. In lung cells, circFADS2 overexpression decreased the level of mature miR-15a-5p, but not miR-15a-5p precursor. LPS treatment decreased miR-15a-5p expression and increased circFADS2 level. Cell apoptosis analysis showed that circFADS2 overexpression reduced miR-15a-5p overexpression-induced apoptosis of LPS-treated lung cells.

**Conclusions:**

CircFADS2 is upregulated in sepsis to suppress LPS-induced lung cell apoptosis by inhibiting miR-15a-5p maturation.

## Introduction

Sepsis refers to a severe clinical condition caused by the body’s excessive responses to bacterial, viral, and fungal infections [1]. Other than ICU treatment, there are no effective approaches for severe septic shock [2]. As a consequence, sepsis is correlated with an unacceptably high mortality rate [3]. It is estimated that 1 out 3 deaths in hospitals are at least partially caused by sepsis [4]. Sepsis-caused inflammation and cell injuries bring damages to almost all organs, such as the lung [5, 6]. Acute lung injury or respiratory failure is a devastating complication of sepsis, resulting in death or disabilities even after active treatments [6]. Therefore, the prevention and treatment of lung injury are critical for the recovery of sepsis patients.

Sepsis is essentially a type of inflammatory disease, requiring the involvement of multiple players [7, 8]. With the increased elucidation of the molecular mechanisms of sepsis, some molecular factors, such as ALK, have been proven to be potential targets for developing novel therapies against sepsis and sepsis-induced organ failures, such as targeted therapy to treat sepsis by regulating regulated gene expression [9, 10]. However, to date, effective targets for sepsis targeted therapy remain lacking. Circular RNAs, or circRNAs, are covalently closed single-strand RNA transcripts that participate in human diseases, including sepsis, possibly by regulating gene expression [11, 12], suggesting that they are potential targets for sepsis-targeted therapy. However, the functions of most circRNAs in sepsis have not been elucidated. CircRNA circFADS2 is proven to play a protective role in LPS-induced cell apoptosis, contributing to sepsis [13]. Our preliminary RNA-seq analysis revealed that the altered circFADS2 expression in sepsis is inversely correlated with miR-15a-5p, which may promote pulmonary diseases [14]. Therefore, this study was performed to explore the role of circFADS2 in sepsis and its interaction with miR-15a-5p, with a focus on sepsis-induced lung injury.

## Materials and methods

### Blood extraction

This study enrolled 50 sepsis patients (28 males and 22 females) and 50 healthy controls (28 males and 22 females) at The Seventh Medical Center, PLA General Hospital between May 2018 and May 2020. The age range of sepsis patients and healthy controls was 40–68 years, with a median age of 54. Therefore, these two groups of participants have similar age and gender distributions. All sepsis patients were caused by bacterial infections and excluded if they had other severe clinical disorders and any therapy initiated within 3 months prior to admission. Healthy controls showed normal physiological functions in systemic physiological exams. The study was approved by the Ethics Committee of our hospital. All participants signed the informed consent. Patients were treated with systemic antibiotics. Blood (3 ml) was extracted under fasting conditions from patients and controls on day 1 and day 8 (after treatment for 1 week) after admission.

### Plasma preparation and human bronchial epithelial cells (HBEpCs)

Blood samples were mixed with citric acid to a ratio of 10:1. The mixture was centrifuged at 1300 g for 15 min at room temperature to separate plasma.

HBEpCs from Sigma-Aldrich (USA) were treated with LPS to serve as the sepsis cell model. These cells were cultured in Bronchial Epithelial Cell Medium (Sigma-Aldrich) at 37 °C, 95% humidity, and 5% CO_2_. To perform LPS treatment, HBEpCs were cultured in the medium supplemented with 0, 1, 2, 4, 8, and 10 μg/ml LPS (Sigma-Aldrich) for 48 h prior to the subsequent assays.

### Vectors, miRNA, and transfections

CircFADS2 fragment was amplified using forward primer GGAATTCATGGGG AAGGGAGGGAACCA and reverse primer ATAAGAATGCGGCCGCTTCATTTGT GAAGGTAGGCG and inserted into pcDNA3.1(+) CircRNA Mini Vector (#60648, Addgene) to construct CircFADS2 expression vector. Mimic of miR-15a-5p and miRNA negative control (NC) were the products of Sigma-Aldrich. HBEpCs were subjected to transfections with either 1 μg circFADS2 expression vector or 45 nM miR-15a-5p mimic using lipofectamine 2000 (Invitrogen). NC experiments were performed by transfecting empty vector or miRNA NC into HBEpCs, and untransfected cells were included as control (C) cells. Fresh medium was used to culture HBEpCs for 48 h prior to the subsequent assays.

### RNA samples

Plasma samples and HBEpCs were subjected to RNA isolation using Trizol (Invitrogen). At 37 °C, RNA samples were digested for 2 h with DNase I (Invitrogen) to remove genomic DNA. RNA integrity was analyzed by electrophoresis (5% urea-PAGE gel). RNA samples with an OD 260 nm/280 nm ratio close to 2.0 (pure RNA) were used in subsequent experiments.

### RT-qPCR analysis

The preparation of cDNA samples was performed using SS-RT-III system (Invitrogen). All-in-One qPCR Mix (BioCat GmbH) was used to perform all qPCRs with GAPDH as the internal control to analyze circFADS2 expression.

MiR-15a-5p precursor expression was analyzed by All-in-One™ miRNA qRT-PCR reagent kit (GeneCopoeia) with sequence-specific primers for both reverse transcription (RT) and qPCR. The same kit was used to analyze mature miR-15a-5p expression with U6 as the internal control after poly(A) addition and poly(T) as the reverse primer for both RT and qPCR. The sequence specific primers are circFADS2 forward ATGGGGAAGGGAGGGAACCA and reverse TTCATTTGTGAAGGTAGG CG, GAPDH forward GCACCGTCAAGCTGAGAAC and reverse GGTGAAGACGC CAGTGGA, miR-15a-5p precursor forward ATCCAGTGCGTGTCGTG and reverse TGCTTAGCAGCACATAATG, mature miR-15a-5p forward TAGCAGCACATAATG GTTTGTG and reverse CTCAACTGGTGTCGTGGA, and U6 forward CTCGCTTC GGCAGCACA and reverse ACGCTTCACGAATTTGCGTGTC. Each PCR was performed in three technical replicates, and the 2^-ΔΔCT^ method was used for the Ct normalization to the corresponding controls.

### Cell apoptosis analysis

HBEpC apoptosis was analyzed at 48 h after cell transfection. In brief, 2 ml medium containing 20,000 cells was transferred to each well of 6-well plates. After the addition of 10 μg/ml LPS, cells were cultured at 37 °C for 48 h, followed by three washes with pre-cold PBS. Cells were then resuspended in binding buffers and stained with PI and FITC-annexin V (Sigma-Aldrich) for 15 min. Cell apoptosis was then analyzed by flow cytometry.

### Statistical analysis

Heml 1.0 software was used to plot heatmaps expressing circFADS2 and miR-15a-5p levels in the plasma samples from patients to analyze their expression during treatment. Unpaired t test was used to compare circFADS2 and miR-15a-5p the expression between patients and controls. Mean ± SD values were used to express the in vitro experimental data, and ANOVA Tukey’s test was used for data comparisons. Correlations were analyzed by Pearson’s correlation coefficient. *P* < 0.05 was deemed statistically significant.

## Results

### Altered circFADS2 and miR-15a-5p expression in sepsis is likely induced by LPS

**Fig. 1:**
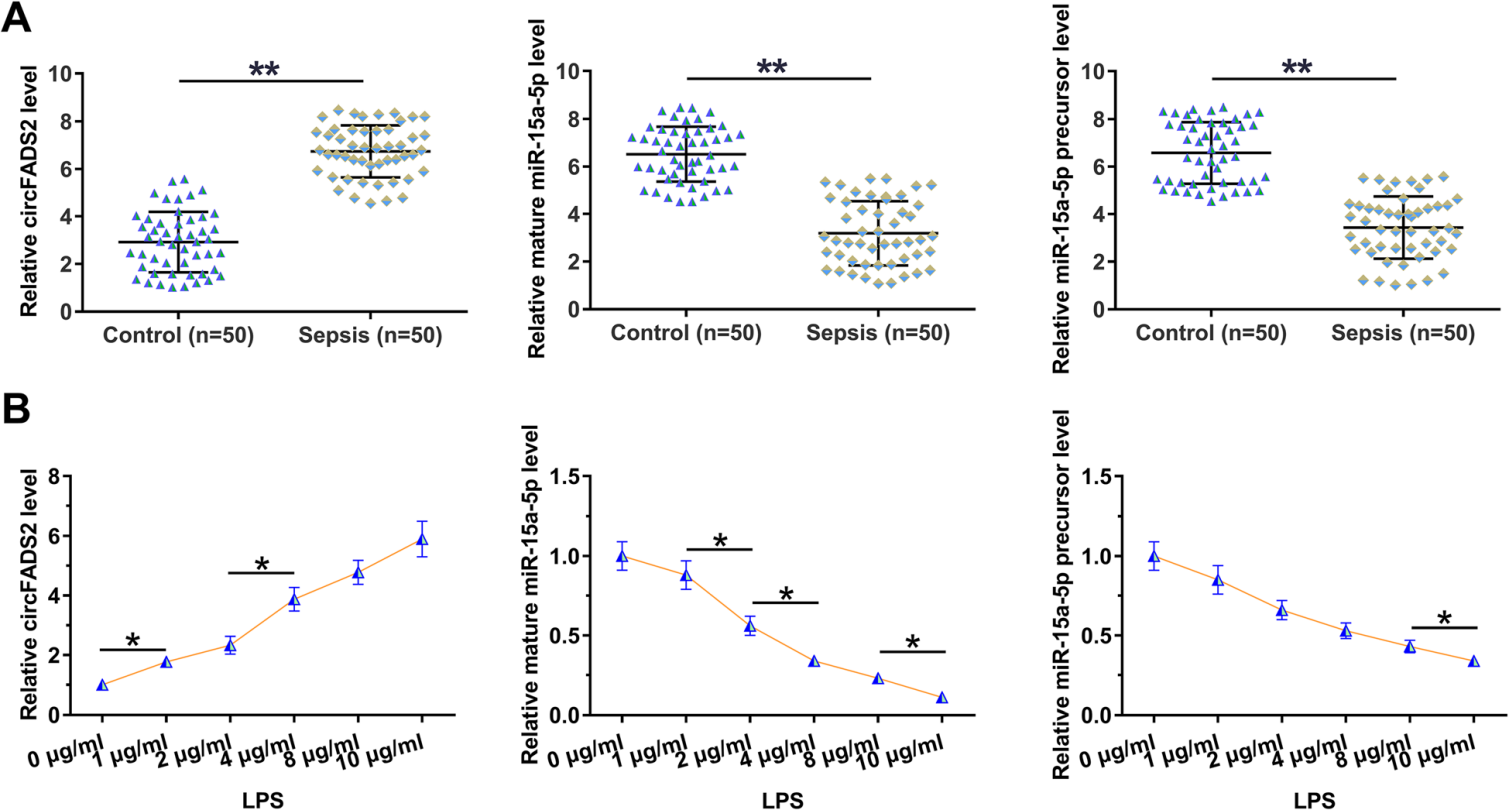
Altered circFADS2 and miR-15a-5p expression in sepsis is likely induced by LPS. Expression of circFADS2, mature miR-15a-5p and miR-15a-5p precursor in plasma samples from sepsis patients (*n* = 50) and healthy controls (*n* = 50) collected prior to treatment was analyzed by RT-qPCR (**a**), **p < 0.01. To test whether the altered expression was caused by LPS, HBEpCs were cultured in the medium supplemented with 0, 1, 2, 4, 8 and 10 μg/ml LPS (Sigma-Aldrich) for 48 h, followed by RT-qPCR to determine gene expression. (**c**), *p < 0.05

Expression of circFADS2, mature miR-15a-5p, and miR-15a-5p precursor in plasma samples from sepsis patients (*n* = 50) and healthy controls (n = 50) collected prior to treatment was analyzed by RT-qPCR. Compared to the controls, circFADS2 was significantly upregulated in sepsis patients, while mature miR-15a-5p and miR-15a-5p precursor were significantly downregulated in sepsis patients (Fig. 1a, *p* < 0.01). To test whether LPS caused the altered expression, HBEpCs were cultured in the medium supplemented with 0, 1, 2, 4, 8 and 10 μg/ml LPS (Sigma-Aldrich) for 48 h, followed by RT-qPCR to determine gene expression. It was observed that LPS treatment increased circFADS2 expression and increased mature miR-15a-5p and miR-15a-5p precursor expression in a dose-dependent manner (Fig. 1b, *p* < 0.05).

### Systemic antibiotics treatment regulated circFADS2 and miR-15a-5p expression

**Fig. 2:**
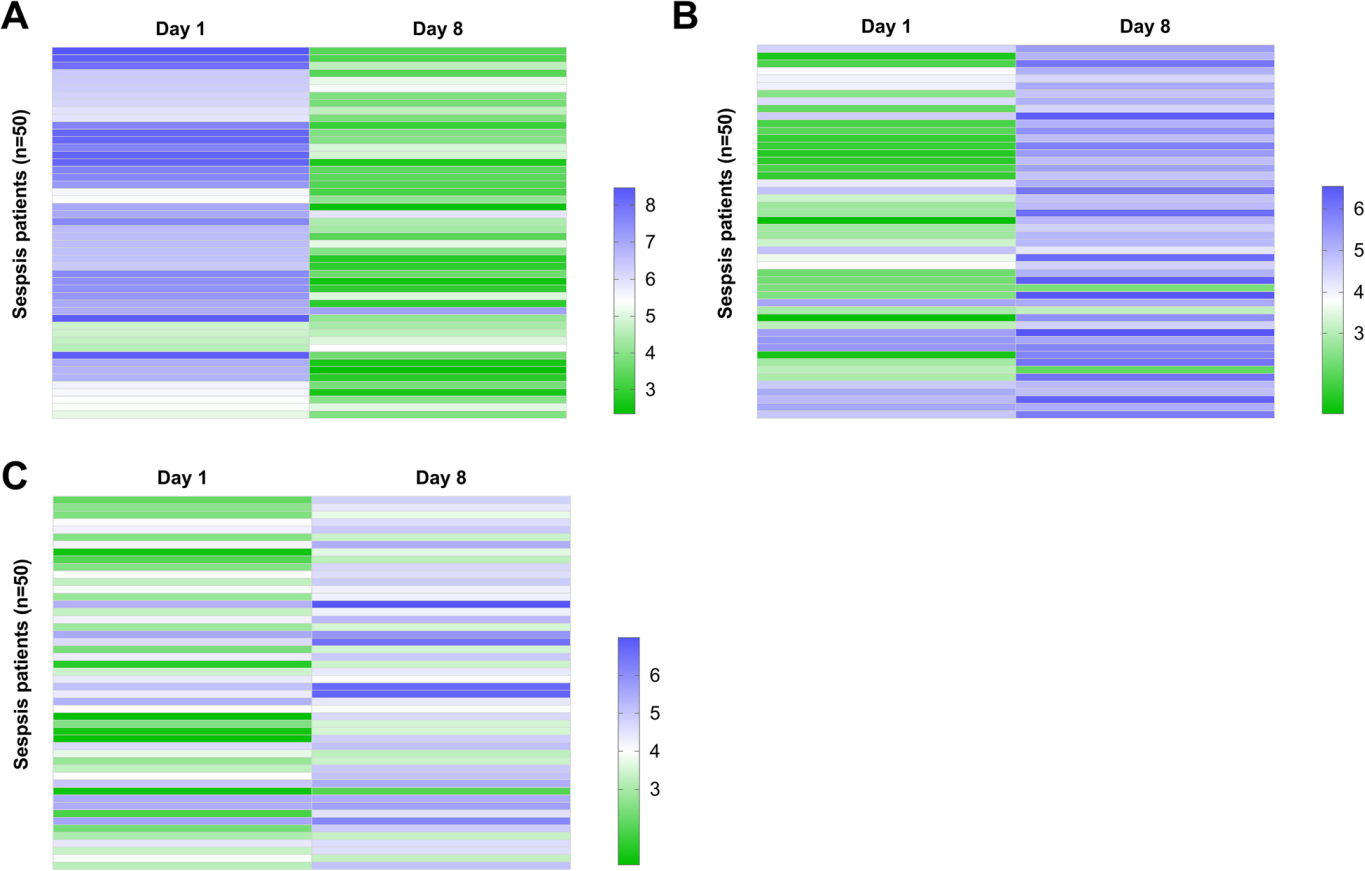
Systemic antibiotics treatment regulated circFADS2 and miR-15a-5p expression. Expression of circFADS2, mature miR-15a-5p and miR-15a-5p precursor in plasma samples of sepsis patients was determined after systemic antibiotics treatment for 1 week (day 8). Heml 1.0 software was used to plot heatmaps expressing the expression of circFADS2 (**a**), mature miR-15a-5p (**b**) and miR-15a-5p precursor (**c**) in plasma samples from patients to analyze their expression during treatment

Expression of circFADS2, mature miR-15a-5p, and miR-15a-5p precursor in plasma samples of sepsis patients was also determined after systemic antibiotics treatment for 1 week (day 8). Heml 1.0 software was used to plot heatmaps expressing circFADS2 and miR-15a-5p expression in plasma samples from patients to analyze their expression during treatment. It was observed that systemic antibiotics treatment downregulated circFADS2 expression (Fig. 2a) and increased mature miR-15a-5p (Fig. 2b) and miR-15a-5p precursor (Fig. 2c) expression in plasma samples of sepsis patients.

### CircFADS2 overexpression suppressed miR-15a-5p maturation in HBEpCs

**Fig. 3:**
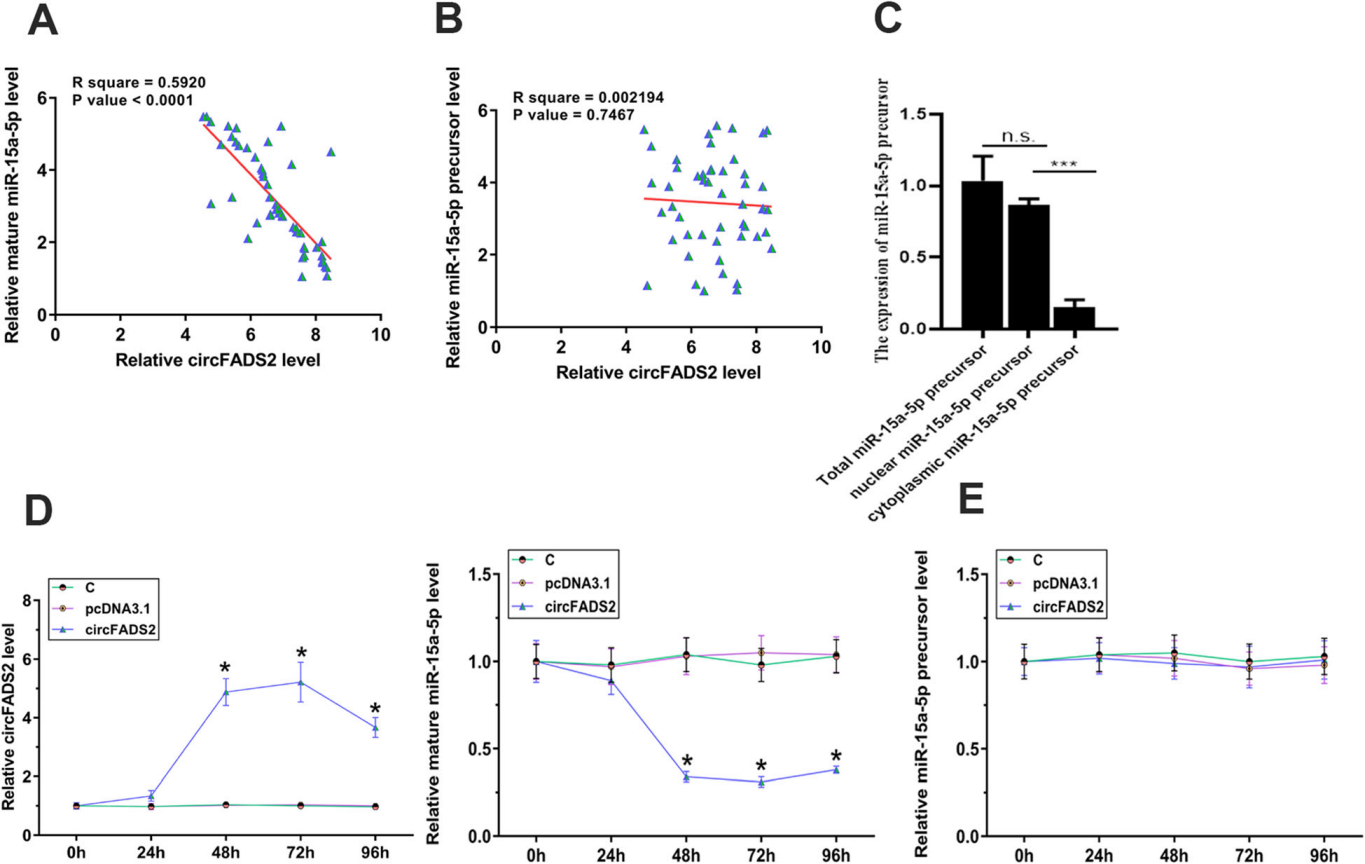
CircFADS2 overexpression suppressed miR-15a-5p maturation in HBEpCs. Pearson’s correlation coefficient analysis was performed to analyze the correlation between expression levels of CircFADS2 and mature miR-15a-5p (**a**) or miR-15a-5p precursor (**b**) across plasma samples from sepsis patients (day 1). MiR-15a-5p precursor and mature miR-15a-5p were detected in the nuclei and cytoplasm, respectively (**c**), ***p < 0.001. CircFADS2 overexpression in circFADS2 expression vector transfected lung cells was confirmed RT-qPCR every 24 h until 96 h (**d**), *p < 0.05. Mature miR-15a-5p and miR-15a-5p precursor levels was measured to explore the role of circFADS2 miR-15a-5p maturation (e), *p < 0.05 compared to C or NC group

Pearson’s correlation coefficient analysis was performed to analyze the correlation between expression circFADS2 and miR-15a-5p levels cross plasma samples from sepsis patients (day 1). It was observed that circFADS2 was inversely correlated with mature miR-15a-5p (Fig. 3a), but not miR-15a-5p precursor (Fig. 3b). The miR-15a-5p precursor was found mainly in the nuclei (Fig. 3c, *p* < 0.001). CircFADS2 overexpression was determined in lung cells transfected with circFADS2 expression vector every 24 h until 96 h. It was observed that circFADS2 overexpression was achieved from 48 h to 96 h (Fig. 3d, *p* < 0.05). The expression levels of mature miR-15a-5p and miR-15a-5p precursor was then measured to study the role of circFADS2 in miR-15a-5p maturation. It was observed that circFADS2 overexpression decreased mature miR-15a-5p expression in HBEpCs, but not miR-15a-5p precursor (Fig. 3e, *p* < 0.05).

### CircFADS2 overexpression suppressed LPS-induced apoptosis of HBEpCs via miR-15a-5p

**Fig. 4:**
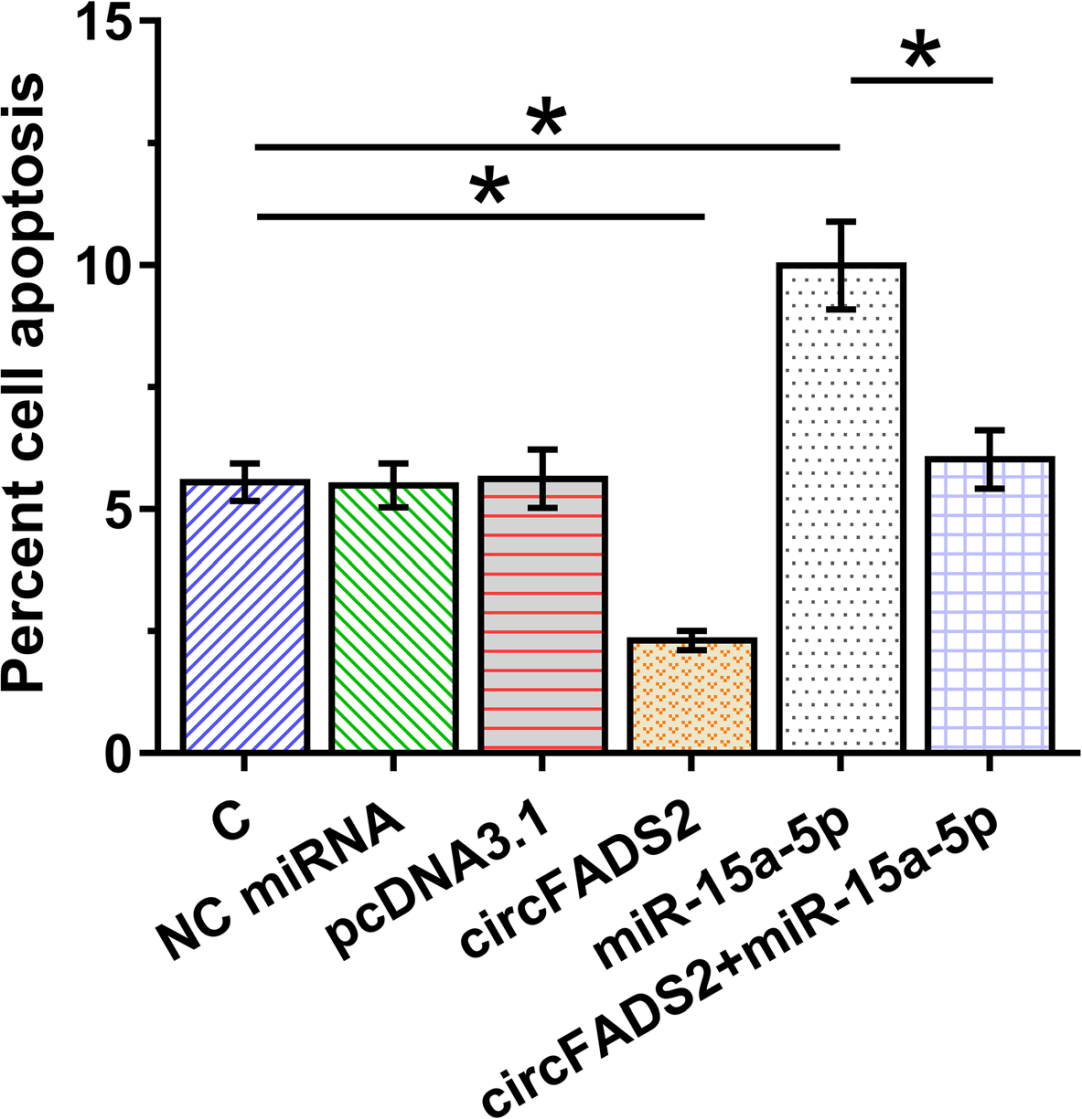
CircFADS2 overexpression suppressed LPS-induced apoptosis of HBEpCs through miR-15a-5p. The role of circFADS2 and miR-15a-5p in regulating HBEpC apoptosis after 10 μg/ml LPS treatment for 48 h was analyzed by cell apoptosis assay, *p < 0.05

The role of circFADS2 and miR-15a-5p in regulating the apoptosis of HBEpCs treated with 10 μg/ml LPS for 48 h was analyzed by cell apoptosis assay. CircFADS2 overexpression decreased cell apoptosis, while miR-15a-5p increased cell apoptosis. In addition, circFADS2 overexpression reduced the enhancement effects of miR-15a-5p overexpression on cell apoptosis (Fig. 4, *p* < 0.05).

## Discussion

This study explored the involvement of circFADS2 in sepsis and studied its potential interactions with miR-15a-5p. We found that circFADS2 was overexpressed in sepsis, and circFADS2 overexpression inhibited miR-15a-5p maturation to suppress LPS-induced by HBEpC apoptosis.

The functionality of circFADS2 has been characterized in cancer biology [15, 16]. It was observed that circFADS2 is overexpressed in lung cancer and could sponge miR-498 to promote cancer cell proliferation and invasion [15]. In addition, circFADS2 is upregulated in colorectal cancer and predicts poor survival of patients [16]. Besides, circFADS2 plays a protective role in LPS-treated chondrocytes by interacting with the miR-498/mTOR axis [17]. In this study, we first showed upregulated circFADS2 in sepsis patients. In addition, LPS treatment increased circFADS2 expression in HBEpCs. Therefore, circFADS2 upregulation in sepsis patients is likely induced by LPS. Interestingly, we showed that circFADS2 suppressed LPS-induced HBEpC apoptosis. In other words, LPS-inducible circFADS2 attenuated LPS-enhanced cell apoptosis, suggesting a feedback regulation between circFADS2 and LPS. Totally, our data showed the protective role of circFADS2 in sepsis.

MiR-15a-5p induces the apoptosis of pulmonary artery smooth muscle cells by interacting with VEGF/p38/MMP-2 signaling pathway [14]. In this study, we showed that miR-15a-5p was downregulated in sepsis, and the downregulation is likely induced by LPS. Moreover, miR-15a-5p overexpression increased LPS-induced HBEpC apoptosis, suggesting that miR-15a-5p also participates in sepsis-induced lung injury.

It has been well established that the function of circRNA in human diseases is to regulate gene expression [11, 12]. Besides, lncRNAs may also sponge miRNAs to suppress their functions. Consistently, circFADS2 sponges miR-498 [17]. Interestingly, our data showed that circFADS2 suppressed miR-15a-5p maturation. However, the mechanism needs to be further elucidated. Whether septicemia symptoms could be alleviated by inhibiting circFADS2 the expression in clinics deserves further investigation.

## Conclusion

CircFADS2 is overexpressed in sepsis, and circFADS2 overexpression reduces miR-15a-5p maturation to suppress LPS-induced of HBEpC apoptosis.

## Data Availability

The data supporting the findings of this study are available on request from the corresponding author. Zhichun Feng, Pediatric Intensive Care Unit, The Seventh Medical Center, PLA General Hospital, No. 5 Nanmencang, Dongshitiao, Dongcheng District, Beijing, 100700, P. R. China. Email: ZhichunFengBeijing@163.com. The data are not publicly available due to containing information that could compromise the privacy of research participants.
